# Green Extraction of Depsidones and Depsides from *Hypogymnia physodes* (L.) Nyl. Using Natural Deep Eutectic Solvents

**DOI:** 10.3390/ijms25105500

**Published:** 2024-05-17

**Authors:** Izabela Baczewska, Maciej Strzemski, Marcin Feldo, Agnieszka Hanaka, Sławomir Dresler

**Affiliations:** 1 Department of Analytical Chemistry, Medical University of Lublin, Chodźki 4a, 20-093 Lublin, Poland; 2 Department of Vascular Surgery, Medical University of Lublin, Staszica 11 St., 20-081 Lublin, Poland; 3 Department of Plant Physiology and Biophysics, Institute of Biological Sciences, Maria Curie-Skłodowska University, 20-033 Lublin, Poland

**Keywords:** lichen, design of experiment, physodalic acid, 3-hydroxhyphysodic acid, physodic acid, atranorin, proline, betaine, lactic acid

## Abstract

Various studies have shown that *Hypogymnia physodes* are a source of many biologically active compounds, including lichen acids. These lichen-specific compounds are characterized by antioxidant, antiproliferative, and antimicrobial properties, and they can be used in the cosmetic and pharmaceutical industries. The main aim of this study was to optimize the composition of natural deep eutectic solvents based on proline or betaine and lactic acid for the extraction of metabolites from *H. physodes*. The design of the experimental method and the response surface approach allowed the optimization of the extraction process of specific lichen metabolites. Based on preliminary research, a multivariate model of the experiment was developed. For optimization, the following parameters were employed in the experiment to confirm the model: a proline/lactic acid/water molar ratio of 1:2:2. Such a mixture allowed the efficient extraction of three depsidones (i.e., physodic acid, physodalic acid, 3-hydroyphysodic acid) and one depside (i.e., atranorin). The developed composition of the solvent mixtures ensured good efficiency when extracting the metabolites from the thallus of *H. physodes* with high antioxidant properties.

## 1. Introduction

Lichens are obligate symbionts between the cells of a fungus (typically belonging to Ascomycota) and a photobiont (usually derived from algae or green algae) [1]. These organisms are the source of approximately 1000 unique bioactive substances that are not synthesized by higher plants [2]. The compounds found in lichens are characterized by a wide range of biological activity, including antibiotic, antibacterial, antiviral, and antifungal properties [3]. Additionally, some lichen metabolites exhibit a positive effect on the proliferation of skin cells and the treatment of skin inflammation [4,5].

*Hypogymnia physode* is a lichen belonging to a species with a high content of specialized lichen metabolites [3,6]. The most important group of metabolites present in *H. physodes* are phenols, which are biosynthesised via the acetyl–polymalonyl pathway [7]. The species produces at least two main classes of phenolic compounds, including depsidones (physodic acid, physodalic acid, 3-hydroyphysodic acid) and depsides (atranorin). These compounds consist of two or three aromatic rings of orcinol or β-orcinol linked by an ester linkage (despides) or an additional ether linkage (depsidones) [7].

The extraction of active compounds is one of the greatest current challenges in the profitable exploitation of specific lichen metabolites [8,9]. Traditional methods for extracting these substances involve using toxic organic solvents, such as acetone, ethyl acetate, and methanol [9]. These methods are not compatible with green chemistry, and the resultant extracts cannot be used directly in the cosmetic or pharmaceutical industries [9,10]. Recently, more researchers have been attempting to implement environmentally friendly methods in the extraction process [8]. These advances are based both on optimizing the physical parameters of extraction using methods such as microwave heating, ultrasound assistance, and pressurized operation (pressurized liquid extraction, supercritical carbon dioxide) and when searching for new non-toxic solvents such as natural deep eutectic solvents (NADES) [8]. NADES are eco-friendly solvents composed of typical plant metabolites such as amino acids, carbohydrates, or natural organic acids [11]. NADES can be defined as a mixture of two or more substances that melt to form a liquid at a certain molar ratio and temperature, with hydrogen bonds forming between the molecules of the compounds. Green extraction using NADES seems to be a favorable method for the cosmetics and pharmaceutical industries due to its ease of preparation, low cost, and low risk for workers [12,13]. Some of the most commonly used ingredients in NADES are betaine, proline, and lactic acid [14,15]. In addition to their ability to form stable NADES, these compounds also possess biologically active properties useful in dermatology. Their many beneficial effects on hair and skin include increasing water retention in cells and collagen building (proline) [16,17]. On this basis, the possibility of extracting lichens with non-toxic solvents for direct use in pharmaceuticals or cosmetics is valuable.

Previously, volatile natural deep eutectic solvents (VNADES) were employed by Kulinowska et al. [9] to extract the lichen *Cladonia uncialis*. The use of lipophilic mixtures of VNADES proved to be more effective at extracting usnic acid than single conventional solvents. However, the presence of skin-irritating phenols may limit the direct use of VNADES extracts in the manufacture of cosmetics or medical products.

The main objective of this work was to optimize the composition of NADES based on proline or betaine and lactic acid for the extraction of metabolites from *H. physodes* using the design of the experimental method and response surface approach. The optimal extraction conditions for maximum extraction efficiency of the four main metabolites present in *H. physodes*, namely physodalic acid, 3-hydroxyphysodic acid, physodic acid, and atranorin, were identified based on the obtained models (Figure S1).

## 2. Results and Discussion

### 2.1. Efficacy of Different Solvents for the Extraction of Specific Metabolites from H. physodes

**Figure 1:**
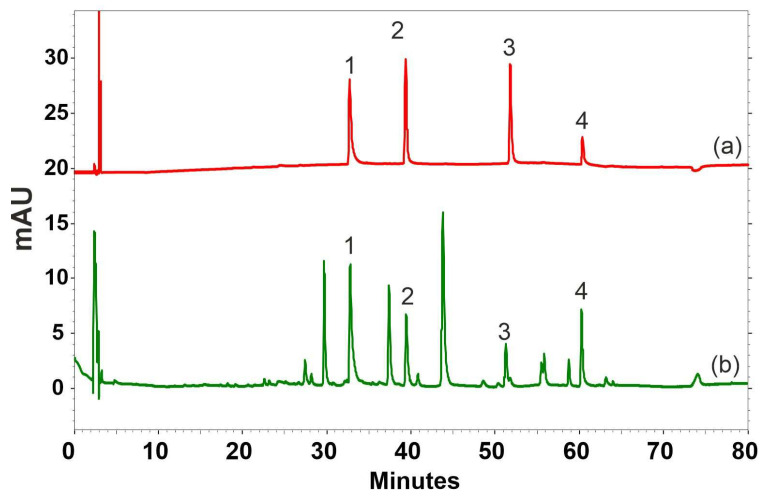
Example chromatograms at 254 nm of standards (**a**) and *Hypogymnia physodes* proline/lactic acid extracts (**b**): (1) physodalic acid, (2) 3-hydroxyphysodic acid, (3) physodic acid, and (4) atranorin.

A characteristic feature of *H. physodes* is its high content of lichen acids, reaching up to 25% depending on the habitat [7]. However, the content of these metabolites usually does not exceed 10–15% of dry weight (DW) [6,18]. High-performance liquid chromatography (HPLC) data indicate that almost 220 mg by a gram of DW was found in the sum of the lichen metabolites assessed based on an exhaustive acetone extraction (Table 1). A previous report identified four compounds (physodalic acid, 3-hydroxhyphysodic acid, physodic acid, and atranorin) as the main active metabolites present in *H. physodes* [6], which is in agreement with the obtained data. The examined raw material contained four main lichen acids, i.e., three depsidones, physodalic acid, 3-hydroxyphysodic acid, physodic acid, and one depside, atranorin (Figure 1), the content of which was 106.2, 48.8, 54.3, and 9.6 mg/g DW, respectively (Table 1). Although other data reported low levels of usnic acid and chloroatranorin may be present at low levels [19], this was not found in our study.

Chemical solvents, such as acetone, chloroform, and ethyl acetate, are generally considered appropriate extrahents for specific lichen compounds [9,20]. Among non-NADES solvents, ethyl acetate was found to be the most efficient in extracting metabolites from *H. physodes* in a single extraction process. This was expected, as a previous report proved that usnic acid dissolves very well in this solvent [9]. Ethyl acetate enabled the extraction of over 91% of the sum of specific lichen metabolites extracted in a 6-fold extraction with acetone. In general, the efficiency of the one-step extraction using non-NADES solvents decreased in the following order: ethyl acetate > dimethyl sulfoxide > methanol > methanol (80%) > acetone (Table 1 and Table 2). In contrast, neither hexane nor water proved to be suitable solvents for the tested metabolites (Table 2). Although previous work indicated the possibility of using hexane in the extraction of atranorin from *Parmotrema saccatilobum* [21], its lack of detection in the studied extractions was surprising despite the low solubility of atranorin in hexane [20].

In the first stage of testing, studying the efficacy of NADES to extract metabolites from *H. physodes*, screening tests were carried out on 18 mixtures that did not contain any toxic components. Although NADES containing choline chloride have been shown to be highly efficient in extracting secondary metabolites from plant raw materials [15], this study only tested components of the mixtures that could be directly used in cosmetics production and were not included in the prohibited constituents list [10].

The extraction efficiencies of the selected NADES were compared to those of single-component solvents under the same conditions, including a liquid-to-solid (L/S) ratio of 100 (µL/mg), an extraction time of 20 min, and a temperature of 40 °C (Table 2). However, a high level of variability in extraction efficiency was observed among the NADES tested. It was found that the NADES tested were able to extract between 22 and over 95 mg of the sum of the four main metabolites from 1 g of raw material of *H. physodes* thalli (9.30% of water content) in a one-step extraction process. These values represented ranged from more than 10 to about 44% of the total sum of metabolites extracted in a 6-fold acetone exhaustive extraction. The relatively lowest recovery of the selected metabolites was obtained using NADES based on proline and citric or malic acids (Table 2). Since the sum of the major metabolites determined in the acetone depletion extraction was about 220 mg, NADES based on citric or malic acid allowed the extraction of 10 to a maximum of 26% of this amount. Mixtures of proline or betaine with urea showed slightly better efficiencies, reaching over 30% of the total metabolite content. Among the solvents evaluated, NADES based on proline or betaine and lactic acid provided the highest recovery rates, including atranorin extraction. For the proline/lactic acid/water mixture (1:2:2 molar ratio), an extraction level of 72 mg/g of the sum of the four metabolites was achieved, which accounted for 33% of the total lichen substances. In this context, we selected NADES based on a combination of proline or betaine and lactic acid for further optimization steps.

### 2.2. Development of Polynomial Regression Models

As mentioned in Section 2.1, extraction rates are highly dependent on analytical and solvent properties. However, other factors, such as the ratio of the solvent to raw material, extraction time, or extraction temperature, can also significantly influence the yield of a metabolite extraction process [22]. In this study, a Box–Behnken design with five replicates at the central point was employed. The five independent factors, including L/S ratio (X_1_—numerical factor), extraction time (X_2_—numerical factor), lactic acid content (X_3_—numerical factor), water content (X_4_—numerical factor), and NADES types such as proline (0)/betaine (1) (X_5_—categorical factor) were tested for the optimization of *H. physodes* metabolite extraction (Table S1). Based on the experiment conducted according to the established experimental design (Table S1), polynomial regression models were developed separately for each metabolite (Table 3). The quadratic models obtained were modified by removing highly insignificant components (factor with *p*-value > 0.1). Four polynomial equation models developed were highly significant, while the non-fit statistics were at an insignificant level (Table 3). Both the determined coefficient *R*^2^ (ranging from 0.62 to 0.96) and the predictive *R*^2^ (ranging from 0.49 to 0.94) were in reasonable agreement with the adjusted *R*^2^ (with differences between coefficients below 0.2). Furthermore, all developed models exhibited high signal-to-noise ratios (Adeq Precion above 4), indicating adequate signal levels and the navigation of the design space.

### 2.3. Effect of Factors on Metabolite Extraction Efficiency Using NADES

**Figure 2:**
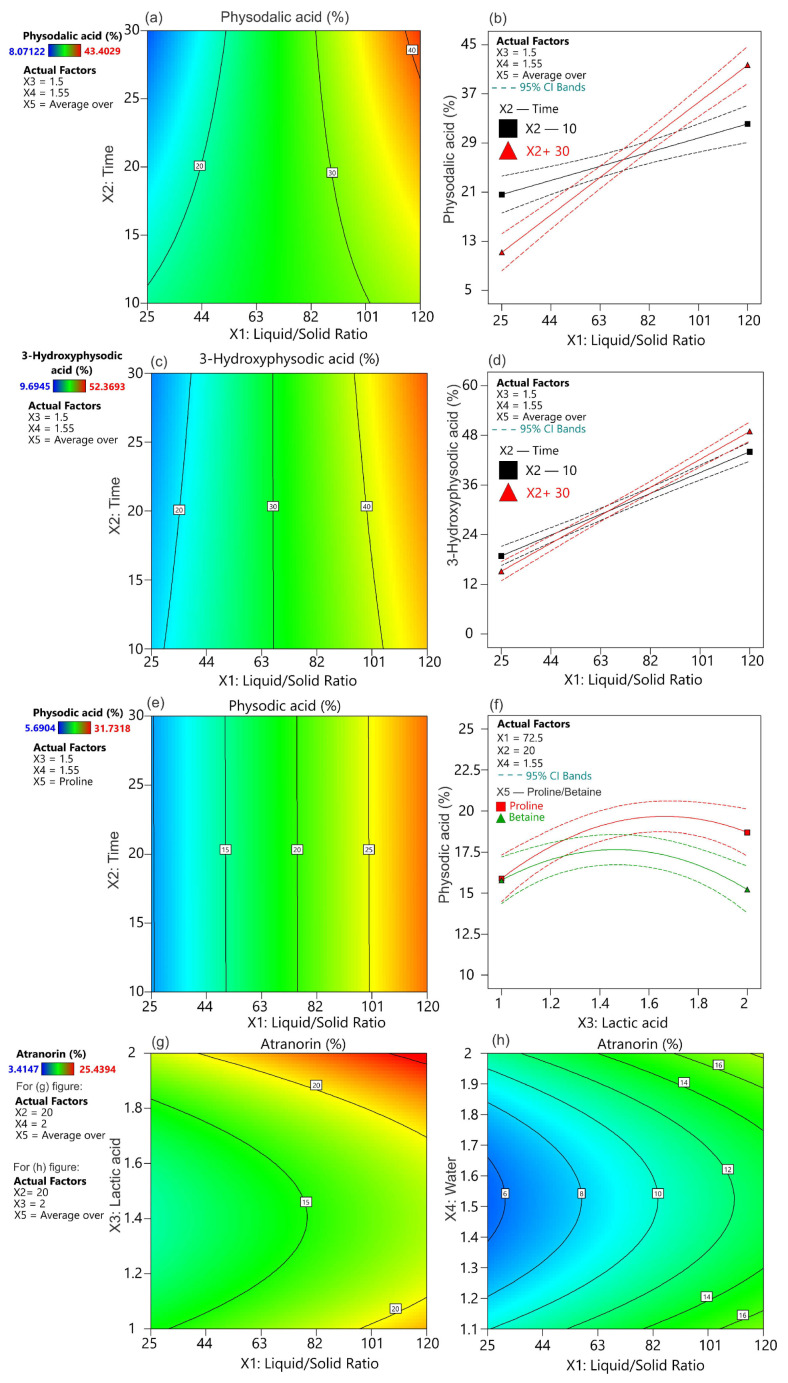
Response of contour plots and interaction plots for the impact of the factors on the extraction rate of *H. physodes* metabolites using NADES: (**a**) the effect of extraction time (X_2_) and liquid-to-solid ratio (X_1_) on physodalic acid extraction yield; (**b**) the interaction effects of extraction time and liquid-to-solid ratio on physodalic adcid extraction yield; (**c**) the effect of extraction time (X_2_) and liquid-to-solid ratio (X_1_) on 3-hydroxyphysodic acid extraction yield; (**d**) the interaction effects of extraction time and liquid-to-solid ratio on 3-hydroxyphysodic acid extraction yield; (**e**) the effect of time extraction (X_2_) and liquid-to-solid ratio (X_1_) on physodic acid extraction yield; (**f**) the interaction effects of lactic acid content (X_3_) and NADES type: proline/betaine (X_5_) on physodic acid extraction yield; (**g**) the effect of lactic acid (X_3_) and liquid-to-solid ratio (X_1_) on atranorin extraction yield; (**h**) the effect of water (X_4_) and liquid-to-solid ratio (X_1_) on atranorin extraction yield.

The L/S ratio is reported to be one of the main factors determining extraction efficiency [9,23]. Indeed, in our study, this factor had a significant effect on the extraction of all metabolites analyzed (Table 3, Figure 2 and Figures S2–S5). The extraction of target compounds increased as the L/S ratio increased from 25:1 to 120:1. This phenomenon was expected and was associated with better contact of the raw material with the solvent [23]. In addition, an increase in the L/S ratio led to a rise in the concentration gradient, which consequently increased the diffusion rate and extraction capacity of the solvent [24].

The extraction time is another important parameter that affects the extraction rate [23]. This finding is closely related to Fick’s second law of diffusion, which states that once the final equilibrium between the material and solvent is reached within a given time, continuing the extraction process is pointless [24]. In addition, excessively prolonging the extraction process can lead to a reduction in substance content due to degradation [25]. The obtained results indicate that extraction durations ranging from 10 to 30 min did not significantly affect the extraction efficiency of the target components (Table 3). This could suggest that the relative equilibrium between the raw material and solvent is established rapidly. A previous study optimizing phenol extraction from oil mixtures using betaine-based NADES observed a rapid time (less than 5 min) to reach equilibrium between the raw material and solvent [26]. However, a significant interaction was found between the extraction time and L/S ratio for physodalic acid and 3-hydroxyphysodic acid (Table 3, Figure 2a–d). It was found that at a low L/S ratio, increasing the extraction time to 30 min resulted in lower extraction efficiencies for both metabolites compared to 10 min. In contrast, at the higher L/S ratio, a longer extraction time (30 min) was required to achieve a high level of extraction efficiency (Figure 2b,d). Such a phenomenon was previously observed during the extraction of usnic acid from *Cladonia uncialis* [9]. It was noted that the equilibrium concentrations of target compounds in the raw material and solvent were expected to be established relatively rapidly at low values of L/S. Conversely, at high L/S ratio values, the high volume of the solvent (distance) must be balanced by extending the extraction time [9].

**Figure 3:**
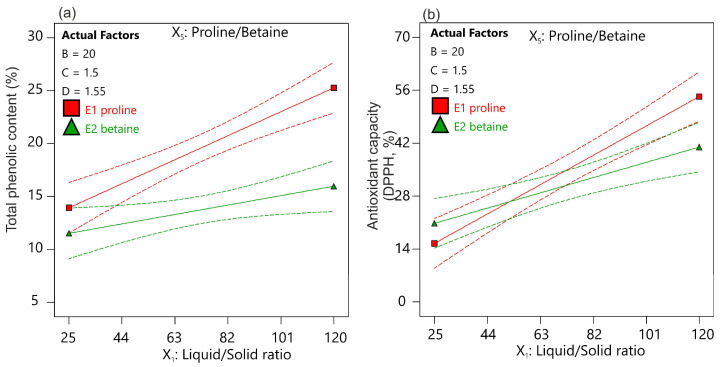
Interaction plots of the impact of the liquid-to-solid ratio (X_1_) and NADES type (X_2_) on antioxidant capacity defined as (**a**) TPC and (**b**) DPPH assays.

The composition of NADESs determines their physical and chemical characteristics, such as viscosity, solubility, and polarities, which greatly influence the efficiency of solvent extraction [15]. The results showed that, except for physodic acid, the molar content of lactic acid was an important factor influencing the extraction efficiency of the other compounds (Table 3). It was found that an increase in lactic acid, while proline or betaine remained constant level, resulted in an increase in extraction efficiency (Table 3, Figure 3). It is worth noting that for physodic acid, there was a significant interaction between the concentration of lactic acid and the type of second component (proline/betaine) (Figure 2f). While no significant effect on the type of NADES was found for the other substances analyzed, physodic acid was extracted more efficiently with NADES based on proline. This effect was only observed when a higher concentration of lactic acid was used. However, no significant linear effect of water content was found (Table 3). This is a surprising observation as water content is known to significantly modify the viscosity of the solvent and the formation of the solvent hydrogen bonding network [27]. The results showed that the square of the water effect was significant (Table 3). The parabolic response reached its maximum in the middle range of water content, except for atranorin (Figures S2–S4). However, the maximum extraction efficiency of atranorin was observed at both maximum and minimum water concentrations (Figure S5). On the one hand, an increase in water content may limit the formation of hydrogen bonds, which could affect the solubility of the analytes. On the other hand, lower water content increases the viscosity and limits solvent dispersion [27].

### 2.4. Determination of Antioxidant Capacity by TPC and DPPH Assays in NADES Extracts

The antioxidant properties of the extracts were determined by two analyzed parameters: total phenolic acids (TPC) and free radical 1,1-diphenyl-2-picrylhydrazyl (DPPH) (Figure 3, Tables S1–S4). Antioxidant capacity has been identified as an important variable that has a significant impact on the pharmaceutical utility of the plant extracts [28]. However, based on the previously confirmed antioxidant properties of the lichen substances [19,29], we assumed that the antioxidant capacity of the extracts would depend on the content of the labeled metabolites. This assumption was confirmed by the calculated correlation coefficients, which ranged from 0.43 to 0.68 and were highly significant (Table S3). Previously, it was demonstrated that the antioxidant capacity of the *H. physodes* extract was only 1.5 times less than that of ascorbic acid [29]. However, since physodic acid exhibits 16 times less antioxidant activity than ascorbic acid, it can be concluded that physodic acid is not the main compound responsible for the antioxidant activity of *H. physode* extracts [29]. Elečko et al. [30] showed that 3-hydroxyphysodic acid is one of the main compounds responsible for the antioxidant activity of lichen extracts. The authors highlight that this compound, due to its possession of a catechol ring fragment with two hydroxyl groups, exhibits exceptionally high antioxidant properties, comparable to ascorbic acid, and several times higher than other specific lichen metabolites [30].

In our study, the TPC content obtained after exhaustive acetone extraction was found to be 129 mg of gallic acid equivalent (GAE) g^−1^ DW, while the antioxidant capacity was 12.2 mg of the Trolox equivalent (TE) g^−1^ DW. The NADES used allowed the extraction of 6 to more than 31% of TPC, and the extracts obtained had antioxidant activity ranging from 12 to 74% (Table S1). The polynomial models developed for both variables, TPC and DPPH, had average coefficients of determination (R^2^) of 0.66 and 0.64, respectively. The antioxidant properties of the extracts, similar to the metabolites, were significantly dependent on the L/S ratio (Table S2). As this parameter increased, the antioxidant capacity of the extracts increased (Figures S6 and S7). However, the effect of this parameter was strongly dependent on the type of NADES component used (Table S2). A significant interaction was found between the two factors, indicating that the increase in the antioxidant capacity of the extracts with a high L/S ratio was significantly greater when proline was used (Table S4). On the other hand, at low L/S ratios, the NADES type had no effect (Figure 3). It was also found that increasing the extraction time resulted in an increase in the antioxidant properties of the extracts. However, for TPC, the effect of time interacted significantly with the L/S ratio. This meant that extended time only resulted in an increase in the antioxidant properties of the extracts when higher values of L/S were used.

### 2.5. Response Prediction and Model Confirmation

Based on the obtained models, the overall desirability [31] was calculated for all variables and separately for proline- and betaine-type NADES (Table 4), as well as for individual responses (Table S4). It was shown that extraction for 30 min using NADES based on proline with a proline/lactic acid/water composition of 1:2:2 and an L/S ratio of 120 (µL/mg) allowed the maximization of all evaluated variables, achieving a desirability value of 0.91 (Table 4). It is considered that desirability values above 0.9 indicate very good properties of the product obtained [32]. In contrast, betaine-based NADES achieved a significantly lower level of desirability—0.765 (Table 4). A higher extraction efficiency with proline-type NADES was found for most variables evaluated individually. Only atranorin was better extracted with betaine-based NADES (Table S4). The obtained optimal extraction parameters (separately for the two types of NADES) were used for the confirmation test. On the basis of the corresponding extraction test, it was found that the experimental and predicted values, except for atranorin extracted from betaine-type NADES, were in good agreement for the analyzed metabolites (with a deviation less than 5) (Table 4). In contrast, the experimental antioxidant values of extracts determined by TPC and DPPH parameters exhibited a poor degree of agreement with the predicted values, ranging from 7 to 25 levels of residue deviation (Table 4).

## 3. Materials and Methods

### 3.1. Chemicals and Reference Standards

The NADES solvent components—L-proline, betaine, lactic acid (90%), urea, malic acid, citric acid; HPLC eluents—used acetonitrile and trifluoroacetic acid; the extraction solvents—dimethyl sulfoxide, acetone, as well as standard—used atranorin were purchased from Sigma-Aldrich (Merck KGA, Darmstadt, Germany). Methanol, ethanol, hexane, and ethyl acetate were supplied by Avantor Performance Materials Poland S.A. (Gliwice Poland). 3-Hydroxyphysodic acid, physodalic acid, and physodic acid were not commercially available. Therefore, these compounds were isolated from *H. physodes* using the following method: 5 g of DW was ground and extracted with 100 mL of acetone for 30 min in an ultrasonic bath. The extraction was repeated twice with a fresh portion of 50 mL of acetone. The combined extracts were passed through a 0.22 µm membrane filter and concentrated to 50 mL using a vacuum evaporator. The main compounds were selected for further collection during several HPLC separations under the conditions described in Section 3.3 and based on the literature [6,33].

### 3.2. Plant Meterials and Extraction

Samples of *Hypogymnia physodes* (Parmeliaceae) were collected in June 2023 in Lublin Voivodeship, Poland (Janów Lubelski town). The lichen was identified based on its morphological and anatomical characteristics by Dr. Hanna Wójciak. The collected samples were manually cleaned and air-dried at 25 °C with air humidity below 30%. The raw material obtained, described in this paper as dry weight (DW), contained 9.30% water (the water was determined by drying the sample portion at 105 C to constant weight). The lichen samples were stored at 4 °C in a sealed glass container prior to further experiments and analyses.

The thallus of *H. physodes* was ground in a mill for 10 min, and 20 mg of each sample was transferred to Eppendorf tubes, then poured with 2 mL of appropriate solution. Both single (1-step) and exhaustive (6-step) extraction were performed. For single extraction, Eppendorf tubes were filled with ground samples of 20 mg each and poured with 2 mL of appropriate solution: (1) the NADES mixture prepared as shown in Table 2 and Table S1 or (2) other solvents (acetone, DMSO, ethanol, ethyl acetate, hexane, 80% methanol, methanol, water) prepared as presented in Table 2. Eppendorf tubes were then placed in an ultrasonic bath at 40 °C for 20 min. Next, the samples were centrifuged at 10,000× *g* for 5 min, and the supernatant was filtered through a 0.22 µm filter (polytetrafluoroethylene membrane, PTFE) prior to analysis. Following the NADES extraction procedures, the supernatants from each extract were quantitatively transferred to vials and diluted with methanol.

For exhaustive extraction with acetone, the procedure of supernatant preparation was the same as described for one-step extraction. After collecting the supernatant, the extraction was repeated five times with a fresh portion of acetone. The supernatant was collected in a separate vessel each time. Immediately after extraction, the samples were subjected to further analysis.

### 3.3. High-Performance Liquid Chromatography

HPLC analyses were performed on a VWR Hitachi Chromaster 600 (Merck, Darmstadt, Germany) coupled with a diode array detector (DAD). The column used was RP18e LiChrospher 100 (Merck, Darmstadt, Germany). The following gradient elution was applied: water with 0.025% trifluoroacetic acid (TFA) (solvent A), acetonitrile (ACN) with 0.025% TFA (solvent B): 0.0–30.0 min A 80%, B 20%; 30.1–60.0 min A 50%, B 50%; 60.1–70.0 min A 25%, B 75%; 70.1–79 min A 0%, B 100%; 79.1–85 min A 80%, B 20%. The recovered fractions were lyophilized using a Christ Alpha 2–4 LDplus lab lyophilizer (Martin Christ Gefriertrocknungsanlagen GmbH, Osterode am Harz, Germany) and were weighed on an analytical balance with a 0.1 mg resolution. Examples of chromatograms of different *H. physodes* extracts are shown in Figure S8.

### 3.4. Determination of Antioxidant Capacity and Total Soluble Phenolic Compounds in Extracts

The total phenolic content (TPC) in the acetone lichen extracts was determined using the Folin–Ciocalteau reagent (Merck, Darmstadt, Germany), following the method previously described by Stasińska-Jakubas et al. [34] TPC values were expressed as the equivalent of mg of gallic acid (Merck, Darmstadt, Germany) per gram of lichen DW.

The method used to estimate the free radical-scavenging activity of the extracts was based on the synthetic free radical 1,1-diphenyl-2-picrylhydrazyl (DPPH, Merck, Darmstadt, Germany) [34]. The results for antioxidative capacity were expressed as the equivalent of mg Trolox (Merck, Darmstadt, Germany) per gram of lichen DW.

### 3.5. Optimising Extraction Variables Using Box–Behnken Design

The Box–Behnken design with five central points was applied for the optimization extraction procedure [35]. Five factors, including four numerical factors (X_1_—liquid-to-solid ratio 25–120 (µL/mg), X_2_—extraction time 10–30 (min), X_3_—lactic acid content 1.0–2.0 (molar ratio value), X_4_—water content 1.1–2.0 (molar ratio value) at three levels and one qualitative factor (X_5_—NADES type: proline/betaine) at two levels were optimized (*n* = 58) (Table S1). Polynomial models were constructed based on the results and supported by ANOVA. The models were validated using calculated fit statistics, including the determination coefficient (R^2^), adjusted R^2^, predicted R^2^, and adequate precision. The optimal extraction parameters were experimentally verified (*n* = 3) to maximize the extraction efficiency. Statistical calculations were performed using Statistica ver. 13.3.03 (Tibco Software Inc., Palo Alto, CA, USA) and Design Expert ver. 13 (Stat-Ease Inc., Minneapolis, MN, USA).

## 4. Conclusions

In this study, an eco-friendly extraction method for specialized metabolites from *H. physodes* was developed using NADES based on proline or betaine and lactic acid. The optimization of four numerical and one categorical extraction factors showed that the optimal extraction conditions were as follows: liquid-to-solid ratio (X_1_)—120 (µL/mg), extraction time (X_2_)—30 (min), lactic acid (X_3_)—2.0 (molar ratio value), water (X_4_)—2.0 (molar ratio value), and proline/betaine (X_5_)—proline. These conditions allowed for the extraction of 40% physodalic acid, 46% 3-hydroxyphysodic acid, 26% physodic acid, and 26% atranorin in a one-step extraction. It is important to note that the extraction efficiency achieved with NADES was similar to that obtained using acetone in the one-step extraction. Both the experimental and predicted values of extraction efficiencies were in good agreement with the confirmatory model test applied. However, the indicators of antioxidant capacity (TPC and DPPH) showed higher volatility and poorer predictive ability.

## Figures and Tables

**Table 1:** Six-step exhaustive acetone extraction of specialized metabolites from *H. physodes*.

Six-Step Exhaustive Acetone Extraction	Physodalic Acid (mg/g DW)	3-Hydroxyphysodic Acid (mg/g DW)	Physodic Acid (mg/g DW)	Atranorin (mg/g DW)
Acetone (1)	49.88 (46.97)	20.08 (41.17)	26.15 (48.19)	5.06 (52.93)
Acetone (2)	29.93 (28.18)	13.18 (27.02)	14.80 (27.28)	2.79 (29.18)
Acetone (3)	11.90 (11.21)	6.87 (14.09)	6.74 (12.28)	0.65 (6.80)
Acetone (4)	6.12 (5.76)	3.49 (7.16)	3.06 (5.64)	0.56 (5.86)
Acetone (5)	5.84 (5.50)	4.09 (8.39)	2.34 (4.31)	0.39 (4.08)
Acetone (6)	2.53 (2.38)	1.06 (2.17)	1.17 (2.16)	0.11 (1.15)
Sum of acetone extracts (1–6)	106.20 (100)	48.77 (100)	54.26 (100)	9.56 (100)

The percentage of extracted metabolites in relation to the total extracted metabolite in the six-step exhaustive acetone extraction is given in brackets.

**Table 2:** Effectiveness of different solvents in the one-step extraction of specialized metabolites from *H. physodes.* The values (± SD) show the mean percentage of extracted metabolite in relation to the total extracted metabolite in the six-step exhaustive acetone extraction.

Non-NADES Solvents	Molar Ratio	Physodalic Acid (%)	3-Hydroxyphysodic Acid (%)		Physodic Acid (%)		Atranorin (%)	
Ethyl acetate	na	85.56 ± 8.969	92.54 ± 3.900		98.49 ± 1.408		107.22 ± 16.203	
Methanol	na	62.30 ± 5.107	72.13 ± 6.547		76.63 ± 5.527		54.18 ± 8.515	
Methanol (80%)	na	46.79 ± 0.820	74.10 ± 0.851		72.30 ± 1.780		13.18 ± 0.544	
Ethanol	na	62.10 ± 3.068	67.21	±4.714	74.60	±6.649	51.67	±2.856
Dimethyl sulfoxide	na	71.54 ± 9.092	76.30	±4.113	82.14	±11.386	98.74	±22.782
Hexane	na	nd	nd		nd		nd	
Water	na	nd	nd		nd		1.57	±0.094
NADES								
Proline/lactic acid/water	1:2:2.5	32.42 ± 4.961	33.83 ± 3.363		25.47 ± 2.479		9.10 ± 3.096	
Proline/lactic acid/water	1:2:2	34.09 ± 2.605	42.96 ± 0.463		25.36 ± 0.851		10.25 ± 0.973	
Proline/urea/water	1:1:2	30.54 ± 5.986	30.08 ± 0.931		18.82 ± 0.610		3.56 ± 0.115	
Proline/urea/water	2:1:6	28.07 ± 2.710	14.95 ± 0.152		9.38 ± 0.083		1.67 ± 0.031	
Proline/malic acid/water	1:1:4	27.15 ± 3.464	34.08 ± 2.116		20.92 ± 1.259		5.13 ± 0.732	
Proline/malic acid/water	1:2:6	10.03 ± 0.222	14.76 ± 0.322		9.36 ± 0.184		1.67 ± 0.105	
Proline/malic acid/water	1:3:4	11.57 ± 0.379	14.74 ± 0.111		9.14 ± 0.074		1.78 ± 0.084	
Proline/malic acid/water	1:2:7	9.98 ± 0.094	14.52 ± 0.199		9.12 ± 0.114		1.67 ± 0.021	
Proline/malic acid/water	1:2:8	10.09 ± 0.265	14.78 ± 0.377		9.31 ± 0.276		1.67 ± 0.042	
Proline/citric acid/water	1:1:8	10.05 ± 0.127	14.62 ± 0.125		9.21 ± 0.070		1.88 ± 0.293	
Proline/citric acid/water	1:2:12	23.12 ± 0.243	32.34 ± 0.572		20.88 ± 0.387		5.96 ± 0.115	
Proline/urea/water	1:1:3	20.09 ± 0.267	29.77 ± 0.597		18.56 ± 0.252		6.07 ± 4.414	
Lactic acid/urea	4:1	11.87 ± 0.643	17.67 ± 1.218		12.77 ± 1.404		3.66 ± 1.004	
Betaine/citric acid/water	1:1.5:9	25.35 ± 0.700	33.18 ± 1.573		21.95 ± 1.290		4.29 ± 0.533	
Betaine/urea/water	1:1:3	33.37 ± 0.984	39.96 ± 0.326		21.53 ± 1.701		1.67 ± 0.188	
Betaine/urea/water	1:1:4	27.19 ± 2.573	41.48 ± 2.620		16.38 ± 0.173		1.57 ± 0.157	
Betaine/urea/water	1:1:5	15.04 ± 1.641	35.88 ± 5.819		12.00 ± 0.262		1.36 ± 0.063	
Bataine/lactic acid/water	1:1:1.4	29.76 ± 5.206	36.50 ± 1.557		20.70 ± 0.644		9.35 ± 0.870	

na—not applicable; nd—not detected.

**Table 3:** Fit statistics, analysis of variance, and regression coefficients of models built for each metabolite (NADES extraction). Variable coded: X_1_—liquid-to-solid ratio, X_2_—extraction time, X_3_—lactic acid content, X_4_—water content, X_5_—NADES type (proline/betaine).

**Physodalic Acid**	***R*^2^ 0.8707**		**Adj *R*^2^ 0.8496**	**Pred *R*^2^ 0.8204**	**Adeq Precision 26.1291**	
			**ANOVA**			
**Component**	**Coefficient**	**Std. Error**	**Source**	**Sum of Squares**	***F*-Value**	***p*-Value**
Intercept	28.04	0.6362	Model	2937.95	41.25	<0.0001
X_1_	10.50	0.6091		2645.22	297.10	<0.0001
X_2_	0.0445	0.6091		0.0475	0.0053	0.9421
X_3_	1.37	0.6091		45.06	5.06	0.0290
X_4_	0.0191	0.6091		0.0087	0.0010	0.9751
X_5_	−0.1085	0.3918		0.6828	0.0767	0.7830
X_1×2_	4.75	1.05		180.43	20.27	<0.0001
(X_3_)^2^	−1.60	0.8031		35.32	3.97	0.0520
(X_4_)^2^	−1.71	0.8031		40.29	4.53	0.0384
			Residual	436.26		
			Lack of Fit	351.07	0.8041	0.7008
			Pure Error	85.19		
			Cor Total	3374.22		
**3-hydroxyphysodic Acid**	***R*^2^ 0.9579**		**Adj *R*^2^ 0.9510**	**Pred *R*^2^ 0.9387**	**Adeq Precision 41.9406**	
			**ANOVA**			
**Component**	**Coefficient**	**Std. Error**	**Source**	**Sum of Squares**	***F*-Value**	***p*-Value**
Intercept	31.53	0.4712	Model	5440.20	139.19	<0.0001
X_1_	14.73	0.4712		5210.36	1066.49	<0.0001
X_2_	0.3013	0.4712		2.18	0.4459	0.5074
X_3_	1.53	0.4712		55.87	11.44	0.0014
X_4_	0.3613	0.4712		3.13	0.6412	0.4272
X_5_	−0.2065	0.2902		2.47	0.5063	0.4801
X_1_X_2_	2.15	0.7815		37.12	7.60	0.0082
(X_3_)^2^	−2.65	0.5949		97.26	19.91	<0.0001
(X_4_)^2^	−1.87	0.5949		48.15	9.86	0.0029
			Residual	239.39		
			Lack of Fit	190.67	0.7635	0.7341
			Pure Error	48.73		
			Cor Total	5679.59		
**Physodic Acid**	***R*^2^ 0.9358**		**Adj *R*^2^ 0.9253**	**Pred *R*^2^ 0.9103**	**Adeq Precision 32.5577**	
			**ANOVA**			
**Component**	**Coefficient**	**Std. Error**	**Source**	**Sum of Squares**	***F*-Value**	***p*-Value**
Intercept	18.54	0.3906	Model	2396.14	89.22	<0.0001
X_1_	9.62	0.3040		2222.06	661.88	<0.0001
X_2_	0.0303	0.3040		0.0220	0.0066	0.9358
X_3_	0.5588	0.3040		7.49	2.23	0.1416
X_4_	−0.5539	0.3040		7.36	2.19	0.1450
X_5_	−0.8978	0.2406		46.75	13.92	0.0005
X_3_X_5_	−0.8406	0.3740		16.96	5.05	0.0291
(X_3_)^2^	−2.15	0.4932		63.64	18.96	<0.0001
(X_4_)^2^	−1.80	0.4932		44.70	13.31	0.0006
			Residual	164.50		
			Lack of Fit	137.90	1.01	0.5412
			Pure Error	26.60		
			Cor Total	2560.65		
**Atranorin**	***R*^2^ 0.6231**		**Adj *R*^2^ 0.5703**	**Pred *R*^2^ 0.4857**	**Adeq Precision 13.9798**	
			**ANOVA**			
**Component**	**Coefficient**	**Std. Error**	**Source**	**Sum of Squares**	***F*-Value**	***p*-Value**
Intercept	9.17		Model	1170.59	11.81	<0.0001
X_1_	3.61			313.22	22.11	<0.0001
X_2_	1.03			25.63	1.81	0.1847
X_3_	2.12			107.99	7.62	0.0080
X_4_	0.5967			8.54	0.6033	0.4410
X_5_	0.8216			39.16	2.76	0.1026
(X_3_)^2^	5.56			426.56	30.12	<0.0001
(X_4_)^2^	4.97			341.54	24.11	<0.0001
			Residual	708.21		
			Lack of Fit	639.13	1.76	0.2017
			Pure Error	69.09		
			Cor Total	1878.81		

**Table 4:** Predicted and experimental mean values at the optimal extraction conditions for proline-based NADES: liquid-to-solid ratio (X_1_)—120 (µL/mg), extraction time (X_2_)—30 (min), lactic acid content (X_3_)—2.0, water content (X_4_)—2.0, proline/betaine (X_5_)—proline; betaine-based NADES: liquid-to-solid ratio (X_1_)—120 (µL/mg), extraction time (X_2_)—30 (min), lactic acid content (X_3_)—2, water content (X_4_)—1.88, proline/betaine (X_5_)—betaine.

Response Variables	Predicted Value	Experimental Value *(n* = 3)	RD (%)	95% PI Low	95% PI High
Proline-based NADES (desirability—0.910)					
Physodalic acid (%)	39.89	39.39	1.269	34.81	44.96
3-Hydroxyphysodic acid (%)	46.29	46.41	−0.259	42.53	50.05
Physodic acid (%)	25.99	27.29	−4.764	23.06	28.92
Atranorin (%)	26.24	25.70	2.101	20.43	32.05
TPC (%)	31.72	29.54	7.380	25.75	37.77
DPPH (%)	69.59	58.85	18.250	55.49	83.68
Betaine-based NADES (desirability—0.765)					
Physodalic acid (%)	40.45	38.98	3.771	35.55	45.35
3-Hydroxyphysodic acid (%)	46.64	47.82	−2.468	43.01	50.27
Physodic acid (%)	23.49	23.60	−0.466	20.68	26.31
Atranorin (%)	25.42	20.75	22.506	19.86	30.99
TPC (%)	19.56	22.23	−12.011	13.72	25.42
DPPH (%)	55.93	44.68	25.179	42.07	69.80

## Data Availability

The data associated with this research can be accessed at https://doi.org/10.5281/zenodo.11189683.

## Supplementary Materials

The following supporting information can be downloaded at: https://www.mdpi.com/article/10.3390/ijms25105500/s1.
