# Supplementary material for: Green Extraction of Depsidones and Depsides from Hypogymnia physodes (L.) Nyl. Using Natural Deep Eutectic Solvents
Source: Int J Mol Sci. 2024 May 17;25(10):5500. doi: 10.3390/ijms25105500 (PMC11121973; doi:10.3390/ijms25105500)
Supplement: Supplementary file 1 [file ijms-25-05500-s001.zip › ijms-2993087-supplementary.pdf]

Supplementary materials

Green Extraction of depsidones and depsides from *Hypogymnia physodes* (L.) Nyl. using Natural Deep Eutectic Solvents

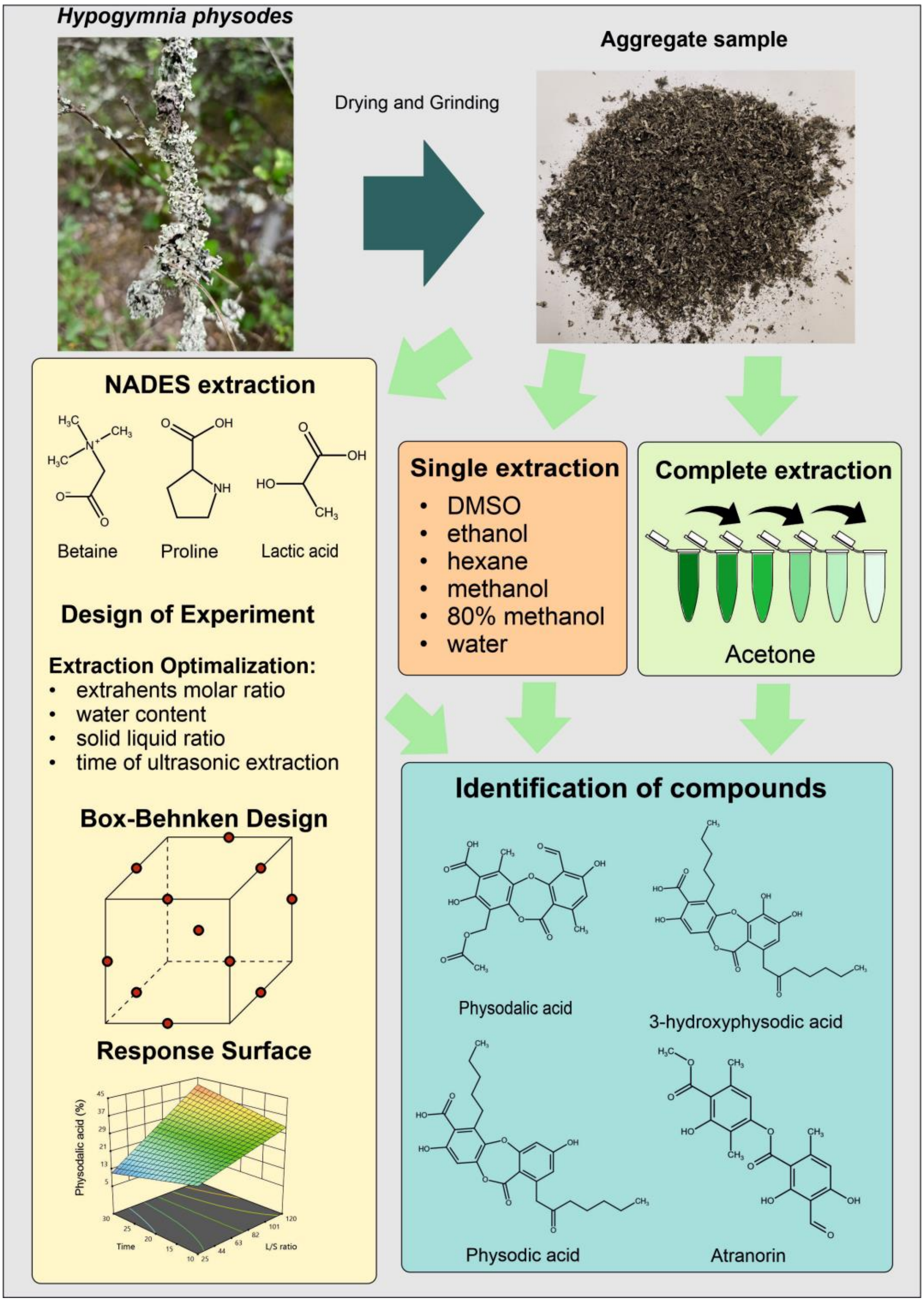

Figure S1. The main steps in the experimental design.

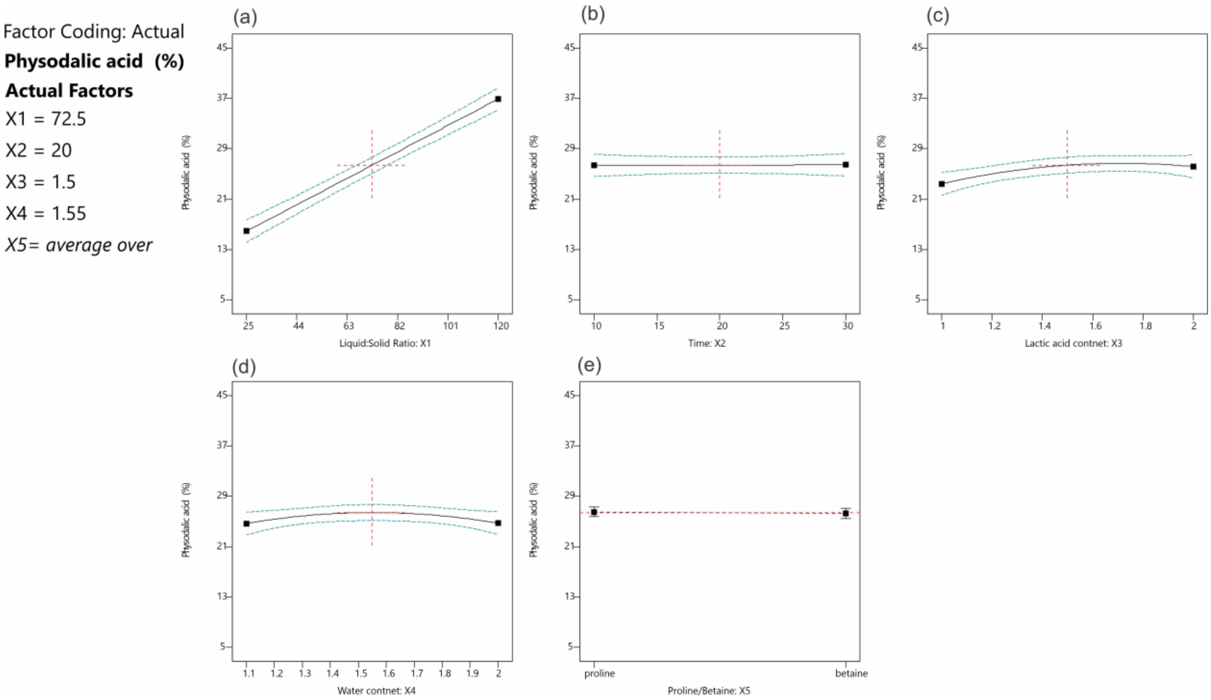

Figure S2. Effect of liquid-to-solid ratio (a), extraction time (b), lactic acid content (c), water content (d) and proline/betaine (e) on physodalic acid extraction efficiency (NADES extraction).

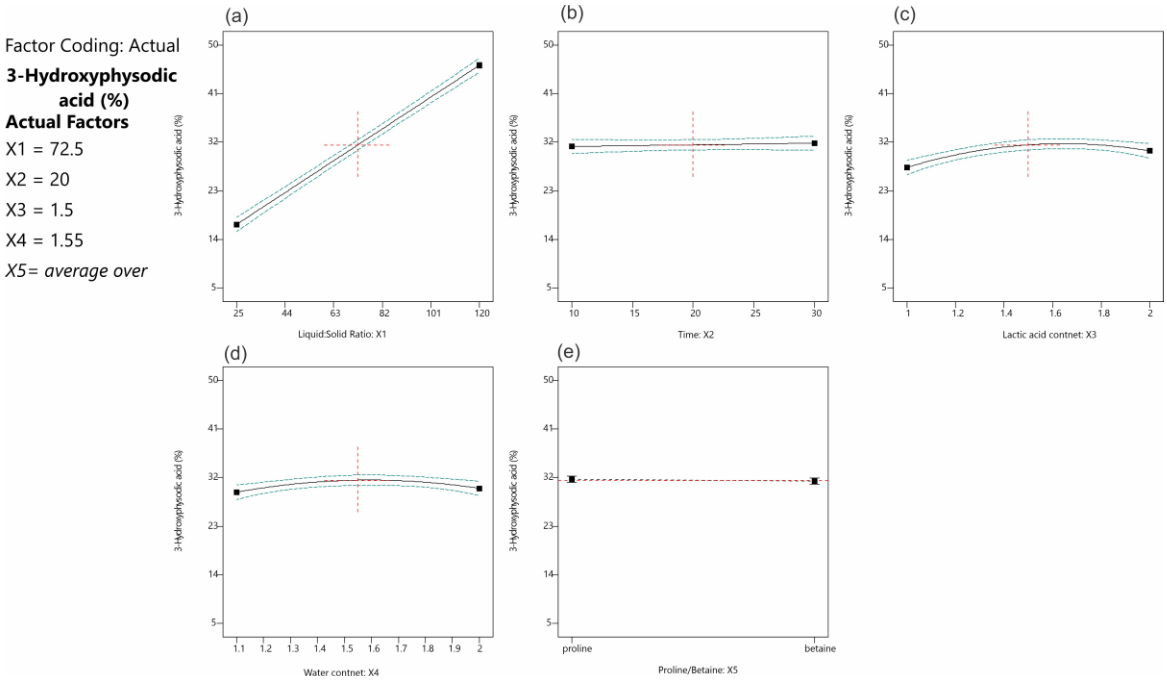

Figure S3. Effect of liquid-to-solid ratio (a), extraction time (b), lactic acid content (c), water content (d) and proline/betaine (e) on 3-hydroxyphysodic acid extraction efficiency (NADES extraction).

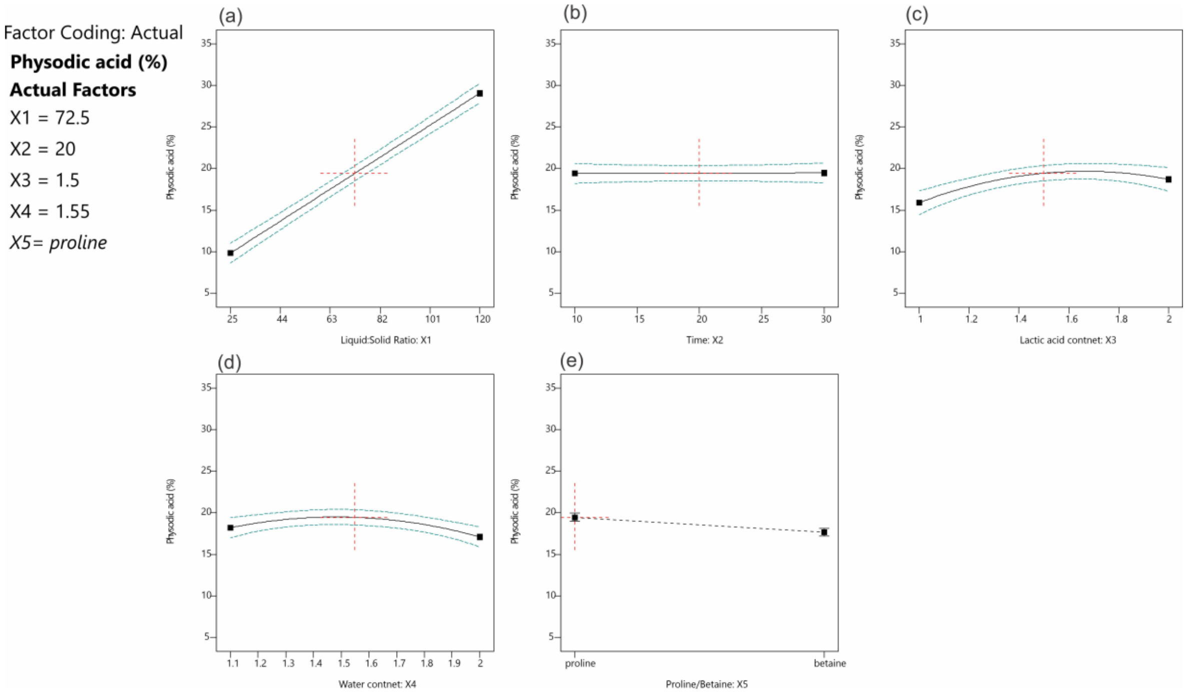

Figure S4. Effect of liquid-to-solid ratio (a), extraction time (b), lactic acid content (c), water content (d) and proline/betaine (e) on physodic acid extraction efficiency (NADES extraction).

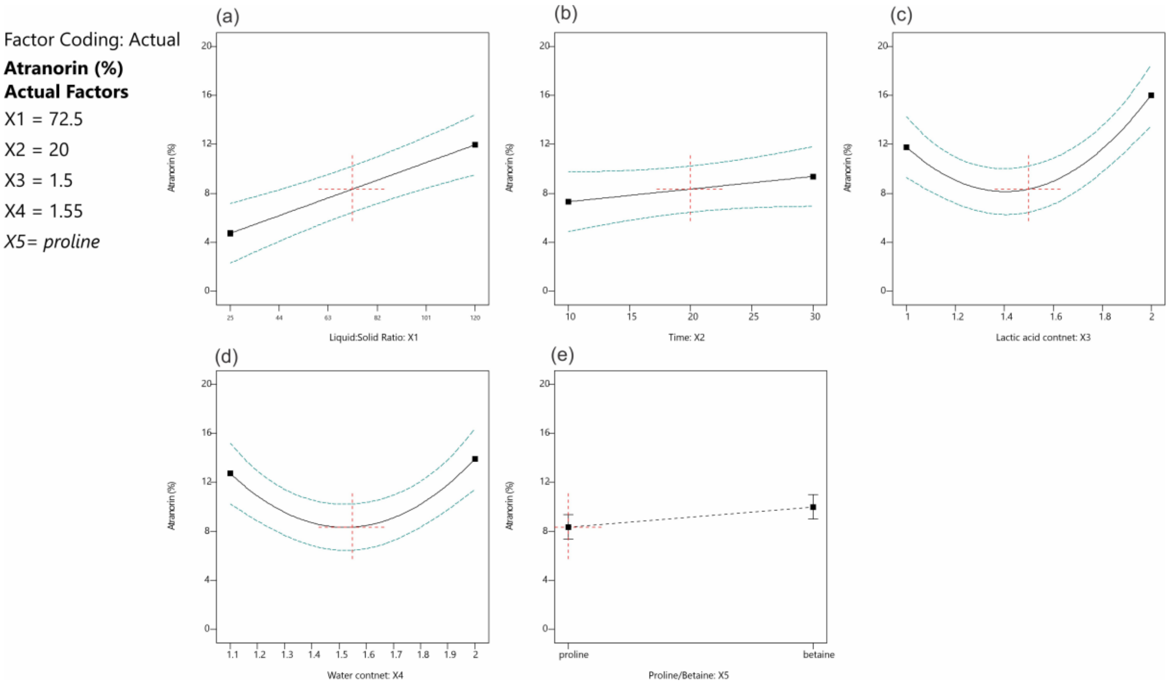

Figure S5. Effect of liquid-to-solid ratio (a), extraction time (b), lactic acid content (c), water content (d) and proline/betaine (e) on atranorin acid extraction efficiency (NADES extraction).

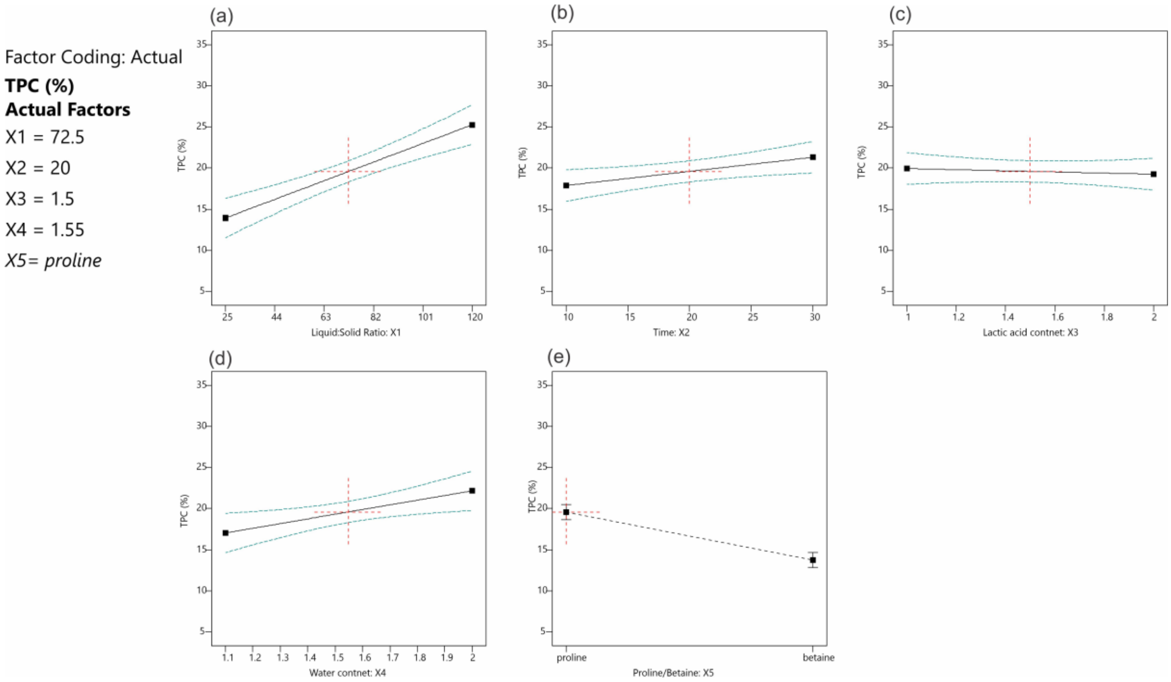

Figure S6. Effect of liquid-to-solid ratio (a), extraction time (b), lactic acid content (c), water content (d) and proline/betaine (e) on total phenolic content (TPC) extraction efficiency (NADES extraction).

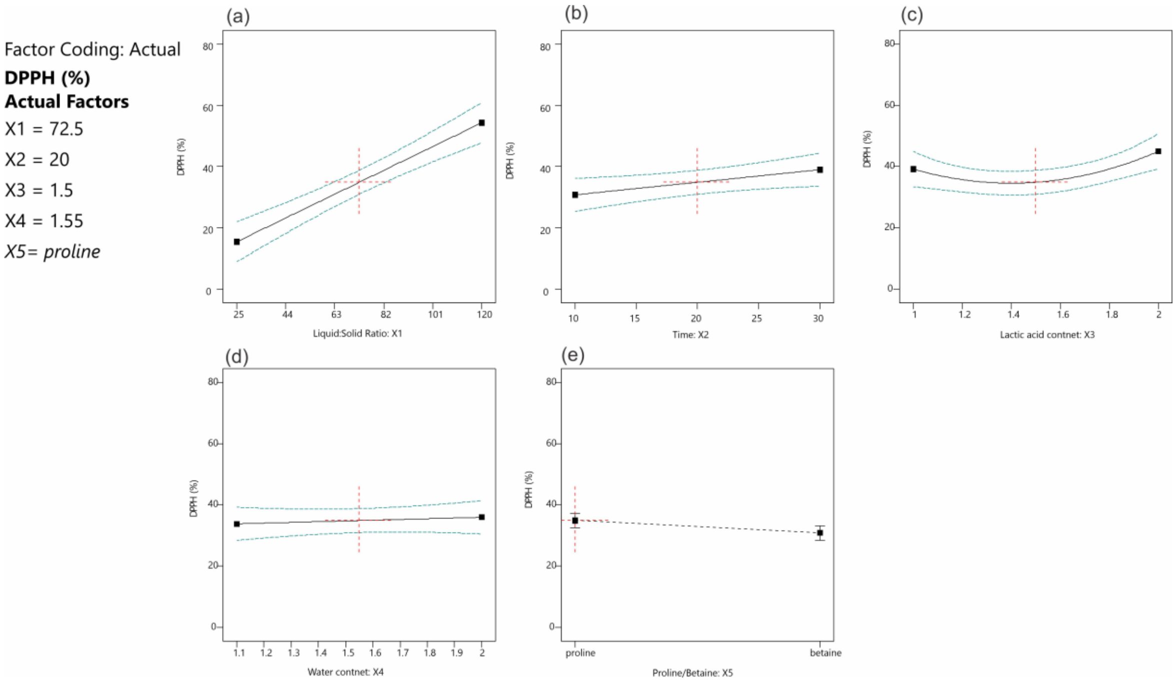

Figure S7. Effect of liquid-to-solid ratio (a), extraction time (b), lactic acid content (c), water content (d) and proline/betaine (e) on antioxidant capacity (DPPH) extraction efficiency (NADES extraction).

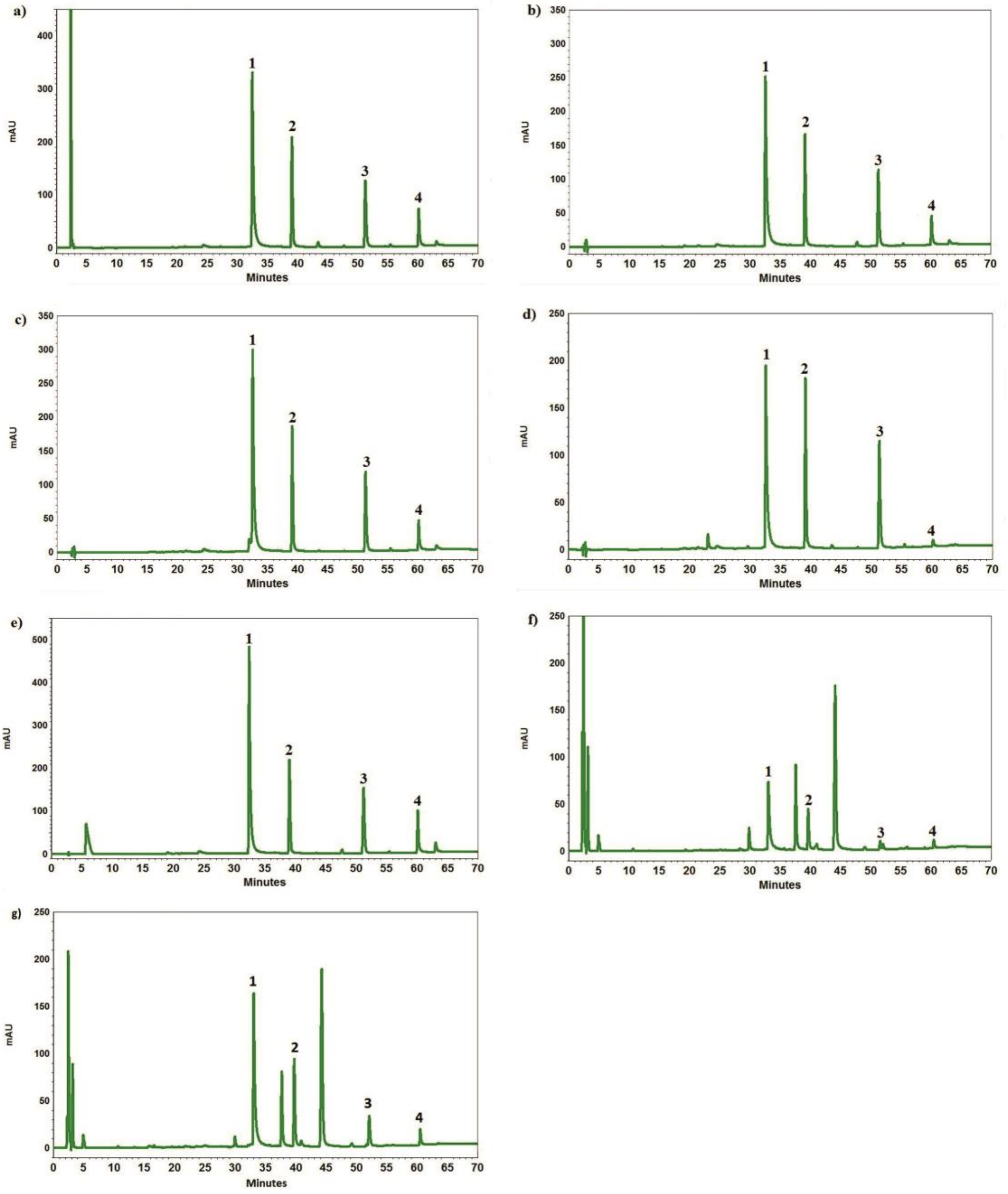

Figure S8. Chromatograms of *H. physodes* extracts (a) DMSO extracts, (b) ethanol extract, (c) methanol extract, (d) methanol (80%) extract, (e) ethyl acetate extract, (f) bataine:lactic acid:water (1:1:2), (g) proline:lactic acid:water (1:1:2); 1 – physodalic acid, 2 – 3-hydroxyphysodic acid, 3 – physodic acid, 4 – atranorin.

Table S1. Factors (type and ratio of extrahents) and responses (metabolites content) (NADES extraction).

Numeric factors				Categoric factors	Responses					
Liquid:Solid Ratio (Volume/Mass) (X_1)	Time (min) (X_2)	Lactic acid content (Molar ratio value) (X_3)	Water content (Molar ratio value) (X_4)	NADES type* (Proline/Betaine) (X_5)	Physodalic acid (%)	3-Hydroxyphysodic acid (%)	Physodic acid (%)	Atranorin (%)	TPC (%)	DPPH (%)
72.5	20	1	1.1	proline	20.7	25.6	13.8	19.1	19.0	31.2
72.5	10	1.5	1.1	proline	22.0	27.9	17.5	6.3	13.2	25.4
25	20	1.5	1.1	proline	8.6	9.6	5.9	3.5	6.0	11.5
120	20	1.5	1.1	proline	35.6	43.5	31.7	16.3	23.1	74.7
72.5	30	1.5	1.1	proline	26.9	32.0	18.1	16.0	20.3	36.1
72.5	20	2	1.1	proline	26.4	29.7	16.4	24.6	22.4	42.7
72.5	10	1	1.55	proline	21.7	26.5	14.6	7.5	17.8	32.0
25	20	1	1.55	proline	18.5	15.0	5.7	12.2	19.2	19.7
120	20	1	1.55	proline	38.9	44.9	24.5	14.0	31.5	60.7
72.5	30	1	1.55	proline	18.7	23.8	13.6	7.6	15.0	29.5
25	10	1.5	1.55	proline	20.3	21.5	13.6	10.1	15.8	26.3
120	10	1.5	1.55	proline	29.7	40.8	29.3	5.2	13.9	41.0
72.5	20	1.5	1.55	proline	25.8	31.6	20.8	9.6	18.5	36.1
25	30	1.5	1.55	proline	16.1	16.4	8.5	11.5	13.7	21.3
120	30	1.5	1.55	proline	40.5	47.2	30.4	12.2	27.4	50.9
72.5	10	2	1.55	proline	23.8	29.7	17.9	19.8	20.4	43.5
25	20	2	1.55	proline	16.2	17.4	9.9	9.5	13.5	24.6
120	20	2	1.55	proline	37.5	44.7	25.8	18.4	27.0	55.8
72.5	30	2	1.55	proline	27.9	32.0	18.1	14.9	22.1	47.6
72.5	20	1	2	proline	22.8	27.9	14.6	16.7	23.5	31.2
72.5	10	1.5	2	proline	30.2	33.6	18.1	10.9	24.1	37.8
25	20	1.5	2	proline	15.0	15.0	6.8	6.2	13.7	20.5
120	20	1.5	2	proline	34.1	44.1	26.3	18.0	27.2	73.0
72.5	30	1.5	2	proline	25.7	30.8	16.0	20.4	26.4	35.3
72.5	20	2	2	proline	22.9	28.7	15.8	19.7	19.8	44.3
72.5	20	1	1.1	betaine	18.9	25.8	18.2	18.4	17.4	55.8
72.5	10	1.5	1.1	betaine	27.8	33.0	16.0	21.4	16.5	31.2
25	20	1.5	1.1	betaine	17.7	16.8	6.3	8.8	12.8	23.8
120	20	1.5	1.1	betaine	38.7	44.9	24.1	17.6	20.3	36.9
72.5	30	1.5	1.1	betaine	23.3	26.5	15.5	9.9	11.4	23.0
72.5	20	2	1.1	betaine	23.1	25.6	15.3	22.9	12.8	33.7
72.5	10	1	1.55	betaine	20.8	25.2	13.8	8.7	9.7	26.3
25	20	1	1.55	betaine	11.8	11.9	7.6	10.0	10.4	25.4
120	20	1	1.55	betaine	32.2	41.2	23.0	15.0	12.5	55.8
72.5	30	1	1.55	betaine	25.0	27.3	19.7	16.6	18.3	34.5
25	10	1.5	1.55	betaine	20.5	14.6	6.8	5.5	8.1	14.0
120	10	1.5	1.55	betaine	33.0	44.7	27.5	11.7	10.6	20.5

72.5	20	1.5	1.55	betaine	26.9	31.4	16.8	10.1	13.1	28.7
25	30	1.5	1.55	betaine	8.1	16.4	6.8	6.5	10.8	18.9
120	30	1.5	1.55	betaine	43.4	52.3	29.5	16.3	18.0	51.7
72.5	10	2	1.55	betaine	26.7	31.2	16.0	13.6	13.7	41.0
25	20	2	1.55	betaine	13.8	14.6	6.3	9.6	10.6	23.0
120	20	2	1.55	betaine	36.0	45.7	24.7	25.4	17.6	50.1
72.5	30	2	1.55	betaine	27.9	30.1	15.7	20.0	15.9	59.1
72.5	20	1	2	betaine	24.4	25.4	13.6	25.1	19.3	44.3
72.5	10	1.5	2	betaine	23.5	28.7	14.9	20.3	12.4	18.9
25	20	1.5	2	betaine	15.0	14.8	5.9	7.0	12.9	19.7
120	20	1.5	2	betaine	33.9	43.1	24.0	16.8	13.4	30.4
72.5	30	1.5	2	betaine	17.6	29.7	15.1	14.0	18.3	49.2
72.5	20	2	2	betaine	25.2	27.7	14.7	23.8	9.5	48.4

Table S2. Fit statistics, analysis of variance, and regression coefficients of models built for total phenolic content (TPC) and antioxidant capacity (DPPH) (NADES extraction). Variable coded: X_1 – liquid-to-solid ratio, X_2 – time, X_3 – lactic acid content, X_4 – water content, X_5 – NADES type (proline/betaine).

**TPC**	R^2 0.6611		Adj R^2 0.6057	Pred R^2 0.5204	Adeq Precision 13.8016	
			ANOVA			
Component	Coefficient	Std. Error	Source	Sum of Squares	*F*-value	*p*-value
Intercept	16.68	0.4556	Model	1150.83	11.95	<0.0001
X_1	3.95	0.7083		374.19	31.08	<0.0001
X_2	1.72	0.7083		70.78	5.88	0.0191
X_3	-0.345	0.7083		2.86	0.2373	0.6283
X_4	1.06	0.7083		26.88	2.23	0.1415
X_5	-2.93	0.4556		498.63	41.41	<0.0001
X_1X_2	2.55	1.23		52.10	4.33	0.0428
X_1X_5	-1.72	0.7083		71.16	5.91	0.0188
X_4X_5	-1.50	0.7083		54.24	4.50	0.0389
			Residual	590.00		
			Lack of Fit	472.23	0.7824	0.7186
			Pure Error	117.77		
			Cor Total	1740.83		
**DPPH**	R^2 0.6421		Adj R^2 0.5920	Pred R^2 0.5075	Adeq Precision 15.7803	
			ANOVA			
Component	Coefficient	Std. Error	Source	Sum of Squares	*F*-value	*p*-value
Intercept	32.87	1.55	Model	7353.74	12.81	<0.0001
X_1	14.77	1.85		5234.28	63.85	<0.0001
X_2	4.11	1.85		404.45	4.93	0.0309
X_3	2.89	1.85		200.49	2.45	0.1242
X_4	1.07	1.85		27.44	0.3347	0.5655
X_5	-2.02	1.19		235.60	2.87	0.0962
X_1X_5	-4.67	1.85		532.04	6.38	0.0148
$(X_3)^2$	7.20	2.41		728.45	8.89	0.0044
			Residual	4098.94		
			Lack of Fit	3685.49	1.70	0.2188

Pure Error	413.46
Cor Total	11452.69

Table S3. Pearson's correlation coefficients between variables (NADES extraction).

Variables	Physodalic acid	3-Hydroxyphysodic acid	Physodic acid	Atranorin	TPC	DPPH
**Physodalic acid**		0.9528 p=0.000	0.8912 p=0.000	0.4190 p=0.001	0.5685 p=0.000	0.6432 p=0.000
**3-Hydroxyphysodic acid**	0.9528 p=0.000		0.9530 p=0.000	0.3917 p=0.002	0.5338 p=0.000	0.6734 p=0.000
**Physodic acid**	0.8912 p=0.000	0.9530 p=0.00		0.2973 p=0.023	0.5181 p=0.000	0.6803 p=0.000
**Atranorin**	0.4190 p=0.001	0.3917 p=0.002	0.2973 p=0.023		0.4303 p=0.001	0.5325 p=0.000
**TPC**	0.5685 p=0.000	0.5338 p=0.000	0.5181 p=0.000	0.4303 p=0.001		0.6342 p=0.000
**DPPH**	0.6432 p=0.000	0.6734 p=0.000	0.6803 p=0.000	0.5325 p=0.000	0.6342 p=0.000	

Table S4. Numeric optimization of extraction parameters to maximize the effect of an individual parameter (NADES extraction). Abbreviation: L/S – liquid-to-solid

**Individual maximising effect**	**Numeric factors**				**Categoric factors**	**Responses**						
	**L/S ratio - X_1**	**Time - X_2**	**Lactic acid - X_3**	**Water - X_4**	**Proline/betaine - X_5**	**Physodalic acid (%)**	**3-Hydroxyphysodic acid (%)**	**Physodic acid (%)**	**Atranorin (%)**	**TPC (%)**	**DPPH (%)**	**Desirability**
**Physodalic acid (%)**	120	30	1.712	1.553	proline	**42.098**	49.094	29.298	14.899	29.419	60.965	0.963
**3-Hydroxyphysodic acid (%)**	120	30	1.644	1.593	proline	42.053	**49.159**	29.251	14.16	29.692	59.956	0.925
**Physodic acid (%)**	120	29.99	1.662	1.482	proline	42.034	49.038	**29.365**	14.287	29.044	59.961	0.909
**Atranorin (%)**	114.828	29.367	1.999	1.102	betaine	37.717	43.194	22.594	**26.176**	19.565	52.702	1.000
**TPC (%)**	119.431	28.419	1.069	1.985	proline	36.927	43.654	23.957	20.311	**31.554**	61.434	1.000
**DPPH (%)**	120	29.996	2	1.971	proline	40.094	46.494	26.251	25.589	31.599	**69.517**	0.918
